# Measurements of Pupillary Diameter and Wavefront Aberrations in Pregnant Women

**DOI:** 10.1155/2016/4129524

**Published:** 2016-02-22

**Authors:** Yesim Altay, Mehmet Metin Altay, Gulizar Demirok, Ozgur Balta, Hulya Bolu

**Affiliations:** ^1^Department of Ophthalmology, Ankara Training and Research Hospital, 06340 Ankara, Turkey; ^2^Etlik Zubeyde Hanim Women's Health Training and Research Hospital, 06010 Ankara, Turkey; ^3^Department of Ophthalmology, Ufuk University Faculty of Medicine, 06520 Ankara, Turkey; ^4^Department of Ophthalmology, Batıgoz Eye Hospital, Izmir, Turkey

## Abstract

*Purpose*. To show whether pregnancy affects the measurements of pupillary diameter and wavefront (WF) aberrations.* Methods*. This was a case-control study including 34 healthy pregnant women in the third trimester and age-matched 34 nonpregnant women. Only women who had no ocular abnormalities and no refractive error were included. We measured photopic and mesopic pupil diameter and WF aberrations at the third trimester and at the second postpartum month. Measurements of the right eyes were used in this study. The differences between groups were analysed by paired *t*-test and *t*-test.* Results*. Pregnant women's mean photopic pupil size in the third trimester was significantly higher than in postpartum period and in control group (3.74 ± 0.77, 3.45 ± 0.53, and 3.49 ± 0.15 mm, *p* < 0.05, resp.). Mesopic pupil size in the third trimester was also higher than in postpartum period and in control group (6.77 ± 0.52, 6.42 ± 0.55, and 6.38 ± 0.21 mm, *p* < 0.05, resp.). RMS-3 and RMS-5 values were higher in pregnancy but these differences were not statistically significant.* Conclusion*. Pregnancy increased photopic and mesopic pupil size significantly but did not increase wavefront aberrations notably. Increased pupil size may be due to increased sympathetic activity during pregnancy. And this activity can be noninvasively determined by measuring pupil size.

## 1. Introduction

Pregnancy results in a tremendous number of changes, both systemic and ocular. Hormonal changes are among the most prominent systemic changes in pregnant women. The placenta, maternal endocrine glands, and foetal adrenal glands combine their productivity to make a high-powered hormone factory [1]. The ocular effects of pregnancy may be divided into physiologic changes, pathologic conditions, or modifications of preexisting conditions [1–3]. Most of the physiologic changes occurring as a result of pregnancy are usually marked in the third trimester, because hormonal activity is at its peak during this period. However these changes are transient and several weeks postpartum, all hormonal activities return to their prepregnant state [4].

Autonomic innervation of the pupil is controlled by both the sympathetic and parasympathetic nerve fibers [5]. Acetylcholine acts on the muscarinic receptors of the sphincter muscle, and so the parasympathetic system leads to constriction of the iris sphincter pupillary muscle and causes pupil size reduction (miosis). Noradrenalin release from the neuromuscular junction (sympathetic system) causes mydriasis. Therefore, the change in pupil size in response to a light stimulus is based on the functional balance between the sympathetic and parasympathetic system [6].

Ocular wavefront (WF) aberration refers to the deviation of light, as it enters the eye compared to optically perfect eye, resulting in blurred images and decreased visual performance [7, 8]. A wavefront is a surface over which an optical disturbance has a constant phase. For light to converge to a perfect point, the wavefront emerging from the optical system must be a perfect sphere centered on the image point. The distance in micrometers between the actual wavefront and the ideal wavefront is the wavefront aberration which is the standard method of showing the aberrations of the eye. Therefore aberrations of the eye are the difference between two surfaces: the ideal and the actual wavefront. Aberrations are subdivided into low order aberrations (LOAs), which can be corrected by sphere-cylindrical lenses, and high order aberrations (HOAs). High order aberrations start at the third level in Zernicke polynomials and they need special design of contact lenses or can be treated by photorefractive surgery. High order aberrations impact vision more severely than LOAs [7]. The pupil diameter is known to be effective on the illuminance and sharpness of retinal image: as the pupil dilates, the retinal image becomes more luminous but the wavefront aberrations tend to increase [9]. OPD-Scan II is a wavefront analyser, corneal topographer, keratometer, autorefractor, and pupilometer all in one unit.

The aim of this case-control study was to investigate whether the pregnancy had an effect on pupil diameter and ocular wavefront aberrations.

## 2. Methods

We examined 34 healthy pregnant women in the third trimester, because most of the physiologic changes occurring as a result of pregnancy are usually marked in the third trimester. Complete ophthalmic examinations including visual acuity and anterior and posterior segment examinations were performed. And only women who had no ocular abnormalities and no refractive error were included in the study. Subjects were excluded if they had a history of systemic or neurologic disorder, intraocular surgery or trauma affecting the pupil, and use of ocular topical or systemic medications. Informed consents were obtained from all patients and tenets of the Declaration of Helsinki were followed. Measurements of photopic and mesopic pupil diameter and ocular wavefront aberrations were recorded at the third trimester, and at the second postpartum month (by then these measurements were expected to return their prepregnant conditions). The right eye and left eye were measured separately in all patients. In present study, only one eye of each subject was assessed to eliminate data duplication bias resulting from symmetricity. So, only the measurements of the right eye of each subject were considered for statistical analyses.

The same measurements were done in age-matched 34 women who have no ocular abnormalities and no refractive error, as a control group.

Photopic and mesopic pupil sizes and WF aberrations were measured using OPD-Scan II Pupillometer/Corneal WF Analyser ARK-10000 system (Nidek, Japan) which cannot measure the scotopic pupil size. For the OPD-Scan II measurements, the subject sat in front of the camera and placed the chin on a chin rest and the forehead against a head band and was instructed to fixate on a target in the center of the camera during the measurement. It uses an infrared detector to capture an image and provides pupillometry measurements. The retina is scanned with an infrared light beam and the reflected light is captured by an array of rotating photodetectors over a 360° area. OPD-Scan automatically performs measurements, first under mesopic conditions followed by one scan under photopic conditions. There is an automated quality-check system which rejects bad measurements. Measurements were performed in the afternoon between 1 and 3 pm, following a rest period of 15 minutes. The pupil camera was used to capture images of each undilated eye in a closed and darkened room under two natural illumination conditions (mesopic: 10 lux; photopic: 100 lux). Quantitative comparisons between different conditions are usually made using root mean square (RMS). To measure RMS for each type of aberration involves squaring the difference between the aberration and mean value and averaging it across the pupil area. In this study, RMS values at 3 mm and 5 mm were recorded simultaneously by OPD-Scan II. RMS expresses the deviation averaged over the entire wavefront in reference to the perfect wavefront. The higher this value is, the higher the levels of ocular wavefront aberrations and irregular astigmatism are within the optical system of the eye which could reduce the visual quality. The majority of normal eyes have RMS-3 and RMS-5 values less than 0.3 *μ*m.

This was a case-control study. Statistical analyses were performed using the Statistical Package for Social Sciences (SPSS) software version 15. The variables were investigated using analytical method (Shapiro-Wilk test) to determine whether or not they are normally distributed. Since the measurements were normally distributed, paired *t*-test and *t*-test were used to compare these variables. We compared the results of pregnant group to the control group, by using *t*-test. In order to show whether any significant change occurred after pregnancy when hormonal levels were assumed to return to their prepregnant state, two months after delivery, the measurements of the pregnant women were compared to postpartum measurements by using paired *t*-test. The results of postpartum group and the control group were also compared with *t*-test. All values were expressed as mean ± standard deviation. A probability value of less than 0.05 was considered statistically significant.

## 3. Results

The mean age of the pregnant women was 28 ± 2.4 years (range: 19–36 years). The mean age of the control group was 27 ± 3.5 years (range: 17–38 years). Visual acuity of each eye was 20/20 in both groups. No anterior segment or posterior segment pathology was recorded.

The mean photopic pupil diameters in the third trimester and in postpartum period were 3.74 ± 0.77 and 3.45 ± 0.53 mm, respectively. It was 3.49 ± 0.15 mm in the control group. A statistically significant difference was found for measurements obtained in the third trimester compared with the postpartum and control group (Table 1). However, there was no significant difference between the measurements of control and postpartum group.

**Figure 1:**
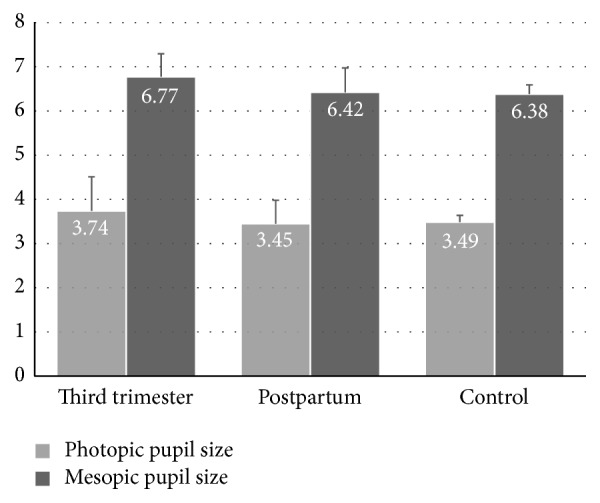
Bar graphics showing comparison of photopic and mesopic pupil sizes of pregnant women at the third trimester and at the second postpartum month to nonpregnant control group of women. Error bars show + standard deviations (SD).

The mean mesopic pupil diameter in the third trimester (6.77 ± 0.52 mm) also decreased (6.42 ± 0.55 mm) in postpartum period. It was 6.38 ± 0.21 mm in the control group. A statistically significant difference was found in the third trimester compared with the measurements of postpartum and control groups (Table 1 and Figure 1). Again, there was no significant difference between the measurements of control and postpartum group.

**Figure 2:**
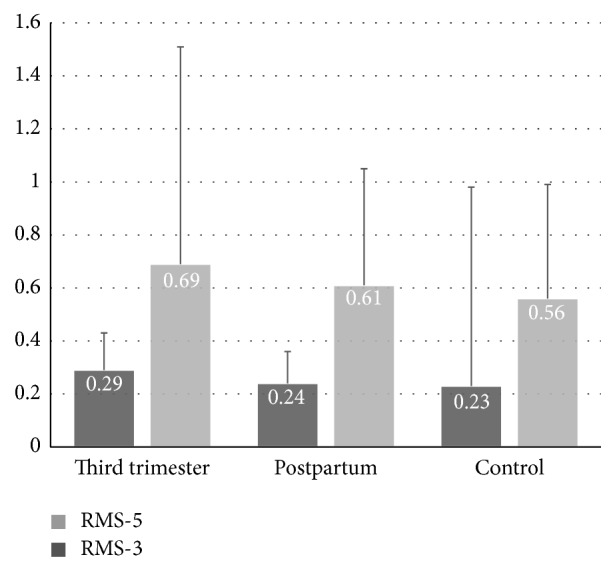
Bar graphics showing comparison of root mean square at 3 mm (RMS-3) and root mean square at 5 mm (RMS-5) values of pregnant women at the third trimester and at the second postpartum month to nonpregnant control group of women. Error bars show + standard deviations (SD).

The mean RMS-3 and RMS-5 were 0.29 ± 0.14 *μ*m and 0.69 ± 0.82 *μ*m in the third-trimester pregnant woman which were decreased to 0.24 ± 0.12 *μ*m and 0.61 ± 0.44 *μ*m in the postpartum period. In the control group these values were 0.23 ± 0.75 *μ*m and 0.56 ± 0.43 *μ*m, respectively. All RMS-3 and RMS-5 values (Figure 2) and other aberrations including coma, trefoil, tetrafoil, spherical, and total high order aberrations were higher in pregnant women but these differences were not statistically significant.

## 4. Discussion

Ocular changes are more common in pregnancy. Because of hormonal influences, pregnancy causes changes in refractive status, cornea sensitivity, visual acuity, and intraocular pressure. However, most of these changes are transient in nature because after several weeks of postpartum period all hormonal activities return to their prepregnant state [10].

One of the most interesting features of the eye is the pupil's reaction to light. Changes in pupil diameter are controlled by two muscles—the dilator and the sphincter—that are differentially influenced by activity in sympathetic and parasympathetic branches of the nervous system. Increased sympathetic activity increases the activity of dilator muscle, prompting dilation, whereas inhibition of parasympathetic activity lessens constriction of the sphincter muscle, which also results in dilation. Thus increases in pupillary diameter can be mediated by activity in either division of the autonomic nervous system [11]. Pupillometric measurements can provide valuable data concerning the functioning of both branches of the autonomic nervous system. Furthermore the pupillometric measurements are a simple and noninvasive technique to obtain information on the autonomic nervous system.

There are several studies which assessed autonomic nervous system (ANS) function by pupillometric measurements. Baum et al. assessed ANS dysfunction in obese children and adolescents by analysis of quantitative pupillography (pupil diameter in darkness), heart rate variability (HRV), and sympathetic skin response (SSR). They demonstrated that both parasympathetic and sympathetic activities are reduced in obesity [12]. Dundaroz et al. evaluated ANS function with pupil diameter measurements in children with enuresis and they reported a decreased parasympathetic activity of the pupillary light reflex [13].

In our study both photopic and mesopic pupil diameter were measured with automated pupillometer. Results showed a significant increase in photopic and mesopic pupil sizes in the third trimester of pregnancy. A mean increase of 0.29 mm in photopic size and a mean increase of 0.35 in mesopic pupil size were recorded. It represents that the iris dilator muscle is activated by sympathetic system more than parasympathetic system.

When we analysed WF aberration values of groups, we found that RMS-3 and RMS-5 values in third trimester were higher than in postpartum period and in the control group. Although the differences between groups were not statistically significant, all other aberrations including coma, trefoil, tetrafoil, and spherical were higher in third trimester. Measurements of higher WF aberrations might have been affected by mesopic pupillary diameter which was larger in third-trimester pregnant women. As is well known the impact of high order aberrations increased with pupil size and the larger pupil size causes greater levels of ocular wavefront aberrations particularly in low lighting conditions [9, 14, 15]. These findings correlate with the knowledge of sympathetic nervous system activation during pregnancy. Kuo et al. reported that autonomic nervous activity showed biphasic changes during pregnancy determined by heart rate variability. Their results indicated that autonomic activity shifted from a lower sympathetic and higher vagal modulation in the first trimester towards higher sympathetic and lower vagal modulation in the third trimester. They concluded that hemodynamic changes of pregnancy and aortocaval compression caused by the growing uterus might be responsible for these changes [16]. Greenwood et al. showed that central sympathetic activity was increased in women with normal pregnancy and was increased even greater in women with hypertensive disorder during the latter months of pregnancy, determined by muscle sympathetic nerve activity (MSNA) [17]. Since MSNA technique requires invasive procedures, evaluating directly vasomotor sympathetic activity in human pregnancy remains a challenging task [18].

Page et al., in an animal study, reported that GABAergic neurons in the paraventricular nucleus (PVN) of hypothalamus which tonically suppress the activity of sympathetic nerves showed less inhibition during pregnancy. There was an increased sympathetic activity in pregnant rats when compared to female virgin rats, determined by microinjection of the GABA antagonist (bicuculline) into the PVN [19].

To the best of our knowledge, this is the first study that describes pupillometric function and ocular wavefront aberration changes that occur during the third trimester of pregnancy.

According to the present study results, pregnancy increased photopic and mesopic pupil size significantly but did not increase wavefront aberrations notably. Therefore we may conclude that there is a sympathetic nervous system activation in pregnancy, and this activation can be noninvasively determined by measuring pupil size.

## Figures and Tables

**Table 1:** Change in photopic and mesopic pupil sizes at the third trimester of pregnancy, at the second postpartum month, and in the control group.

Patient groups	Third trimester mean ± SD	Postpartum mean ± SD	Control group mean ± SD	Third trimester versus control (†) *P*	Postpartum versus control (‡) *P*	Third trimester versus Postpartum (§) *P*
Photopic pupil size (mm)	3.74 ± 0.77	3.45 ± 0.53	3.49 ± 0.15	0.03^*∗*^	0.70	0.04^*∗*^

Mesopic pupil size (mm)	6.77 ± 0.52	6.42 ± 0.55	6.38 ± 0.21	0.0001^*∗*^	0.63	0.005^*∗*^

†, ‡: Student's *t*-test,

§: paired *t*-test,

*∗*: statistically significant difference.
